# Supplementary material for: Lifelong Invertebrate Chordate Central Nervous System Regeneration is Driven by a Migrating Pool of Conserved Neural Stem Cells program
Source: bioRxiv. 2026 Sep 13:2026.09.11.750342. Preprint. [Version 1] doi: 10.64898/2026.09.11.750342 (PMC13622670; doi:10.64898/2026.09.11.750342)
Supplement: Supplementary file 1 [file NIHPP2026.09.11.750342V1-supplement-1.pdf]

bioRxiv preprint doi: https://doi.org/10.64898/2026.09.11.750342; this version posted September 13, 2026. The copyright holder for this preprint (which was not certified by peer review) is the author/funder, who has granted bioRxiv a license to display the preprint in perpetuity. It is made available under a CC-BY 4.0 International license.

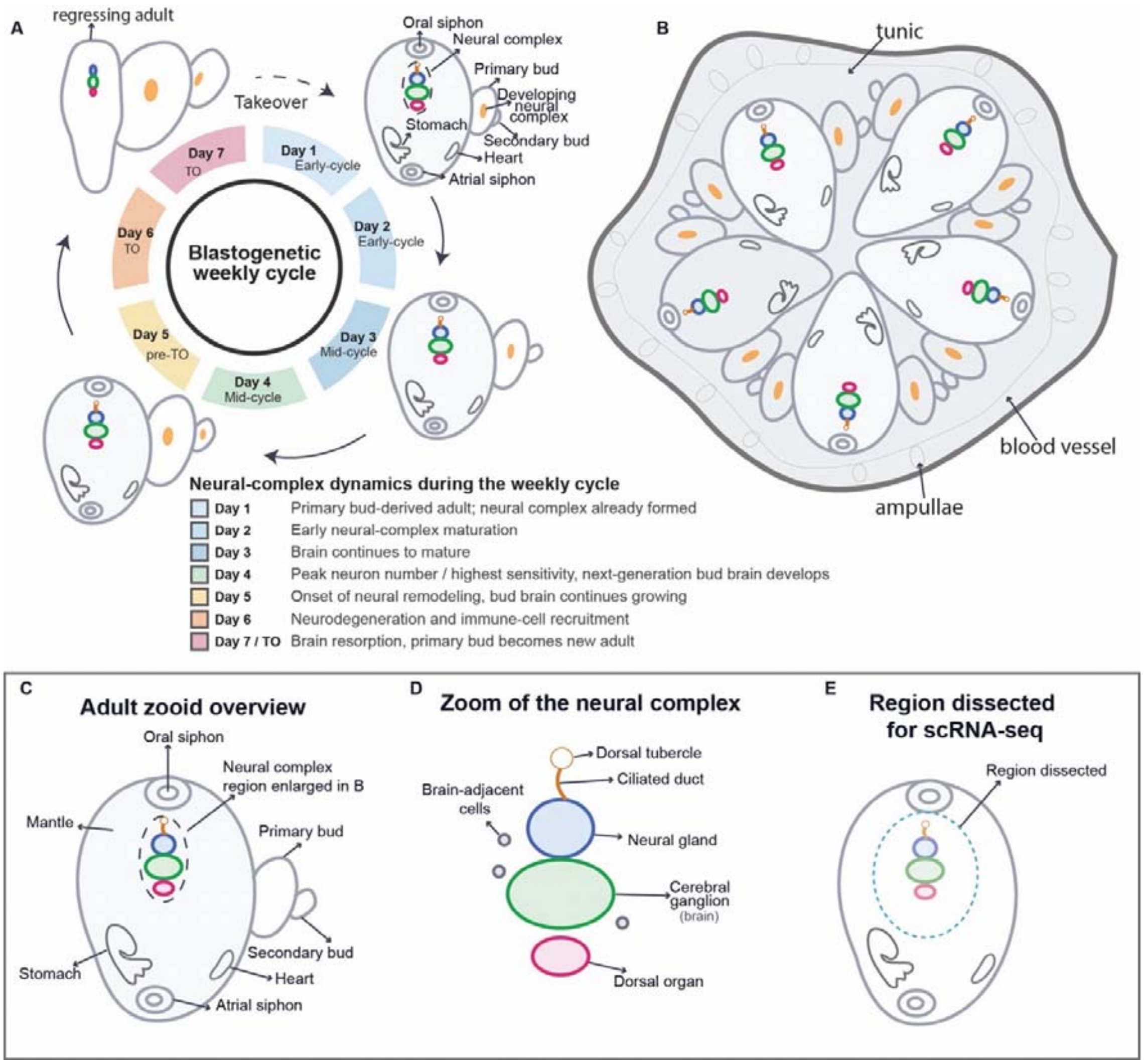

Figure S0. Colony organization, adult zooid anatomy, neural-complex structure, and day-based staging across the weekly blastogenetic cycle of *Botryllus schlosseri*.
(A) *Botryllus schlosseri* reproduces sexually through embryogenesis and asexually through blastogenesis. This schematic illustrates the weekly asexual budding cycle, during which clonally related zooids are generated, mature, regress, and are replaced by the next generation. The dashed arrow between Day 7/takeover and Day 1 indicates a generational transition rather than the continuation of the same adult zooid: at takeover, the regressing adult zooid is resorbed, while the primary bud of the previous cycle becomes the new adult zooid. Thus, the Day 1 neural complex corresponds to the brain that developed in the primary bud during the preceding cycle. During Days 1–3, the adult neural complex continues to mature. Around Day 4, the adult brain reaches maximal neuron number and the zooid shows the highest behavioral sensitivity; at the same time, the neural complex of the next generation is developing in the bud lineage. During Days 5–6, the adult brain enters a remodeling and degeneration phase, characterized by decreasing neuron number, increased apoptotic/neurodegenerative signatures, and increased immunocyte association with the neural complex. At Day 7/takeover, the old adult zooid and its brain are resorbed, while the bud generation replaces it. These stage-dependent neural changes were previously characterized in *B. schlosseri*.
(B) Stylized view of a zooid within the colonial context. The colony is attached to a substrate, covered by a common tunic, and composed of genetically identical zooids produced by asexual budding. Zooids are connected by a shared extracorporeal blood vasculature ending

bioRxiv preprint doi: https://doi.org/10.64898/2026.09.11.750342; this version posted September 13, 2026. The copyright holder for this preprint (which was not certified by peer review) is the author/funder, who has granted bioRxiv a license to display the preprint in perpetuity. It is made available under a CC-BY 4.0 International license.

in peripheral ampullae. This transparent and experimentally accessible vascular network allows live observation and can be used for injection-based manipulation.
(C) Overview of an adult zooid showing the oral siphon, atrial siphon, mantle, stomach, heart, primary bud, secondary bud, and the neural-complex region.
(D) Enlarged schematic of the neural complex, including the cerebral ganglion/brain, neural gland, ciliated duct, dorsal tubercle, dorsal organ, and associated brain-adjacent cells.
(E) Region dissected for Smart-seq3 single-cell RNA-seq, encompassing the neural complex and closely associated tissues.

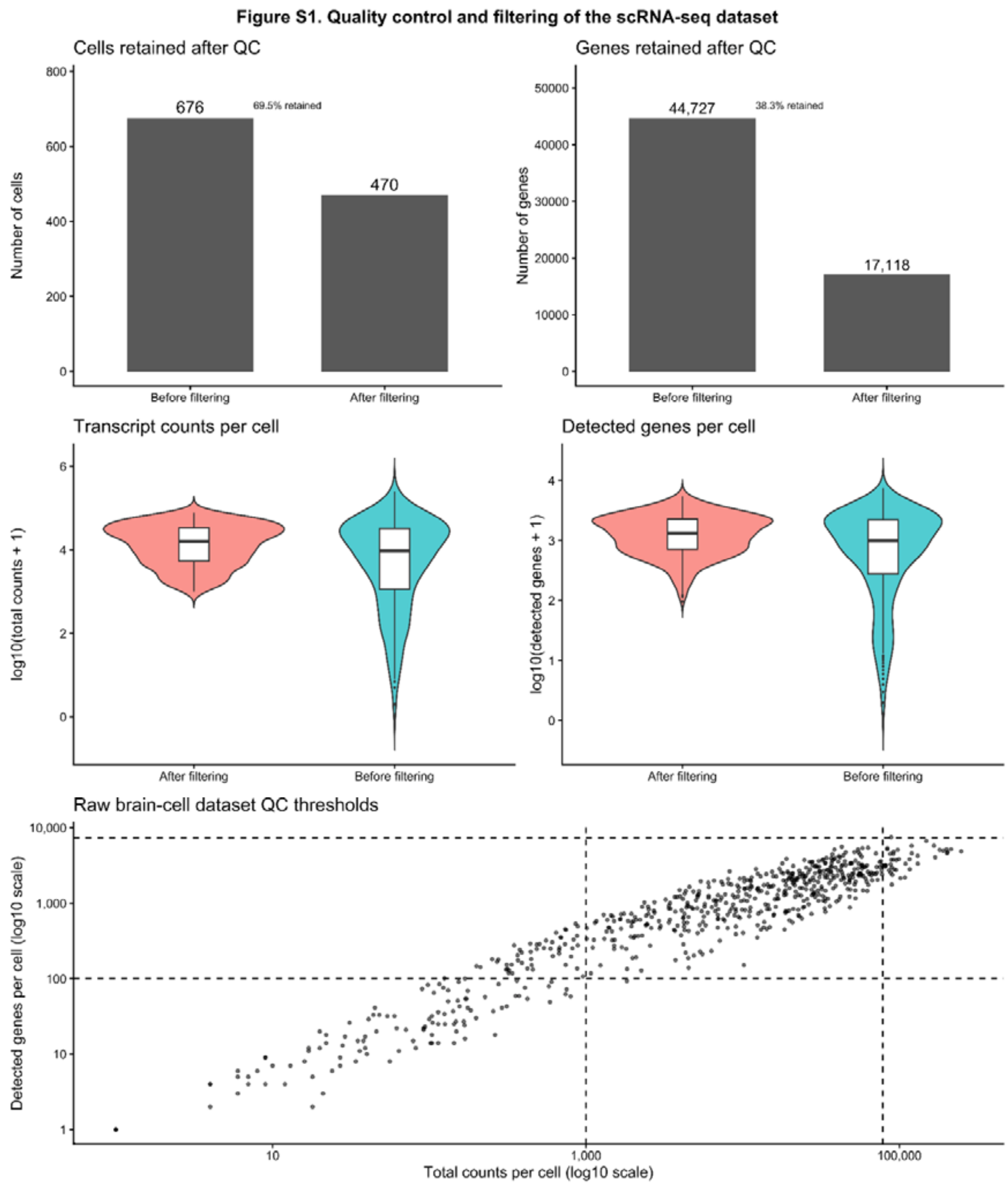

Figure S1. Quality control and filtering of the scRNA-seq dataset.
Summary of the quality-control workflow applied to the Smart-seq3 dataset generated from dissected adult *Botryllus schlosseri* neural complexes. The top panels show the numbers of

bioRxiv preprint doi: https://doi.org/10.64898/2026.09.11.750342; this version posted September 13, 2026. The copyright holder for this preprint (which was not certified by peer review) is the author/funder, who has granted bioRxiv a license to display the preprint in perpetuity. It is made available under a CC-BY 4.0 International license.

cells and genes before and after filtering, indicating retention of 470 of 676 cells and 17,118 of 44,727 genes. The middle panels show the distributions of total transcript counts and detected genes per cell before and after filtering. The bottom panel shows the raw brain-cell dataset in count–gene space, with dashed lines indicating the thresholds used for filtering. Cells were retained using fixed lower cutoffs together with upper thresholds defined from the raw dataset using a median + MAD-based approach. Exact threshold values and summary statistics are reported in the Table.

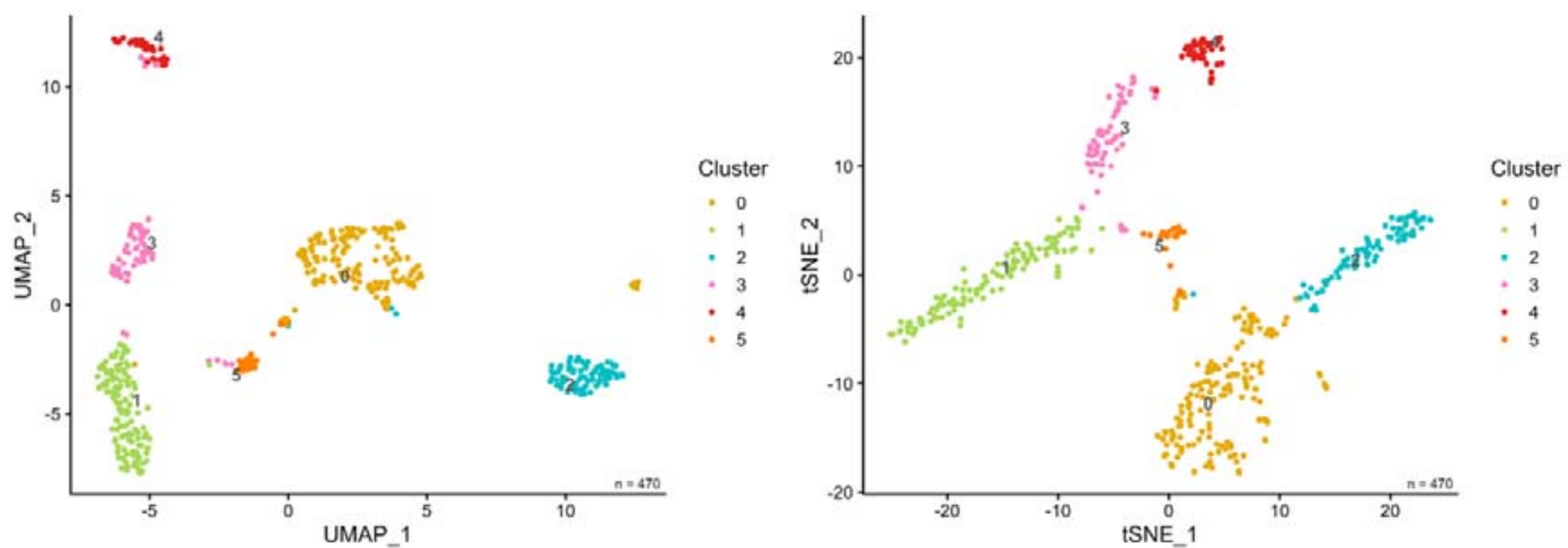

Figure S2. t-SNE embeddings of the filtered single-cell dataset.
t-SNE projections of the filtered neural complex dataset with cells colored by cluster and labeled with the final biological cluster names.

bioRxiv preprint doi: https://doi.org/10.64898/2026.09.11.750342; this version posted September 13, 2026. The copyright holder for this preprint (which was not certified by peer review) is the author/funder, who has granted bioRxiv a license to display the preprint in perpetuity. It is made available under a CC-BY 4.0 International license.

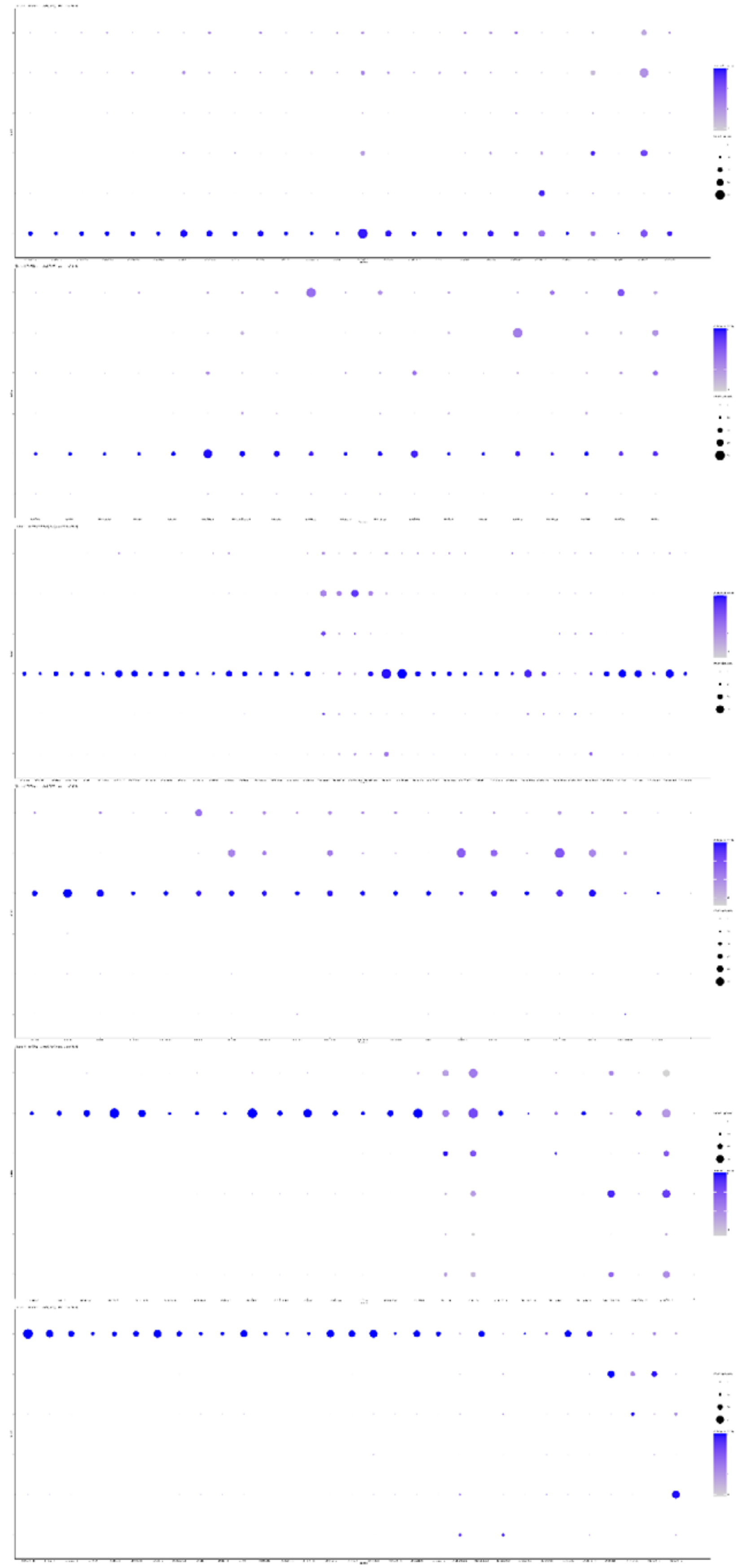

bioRxiv preprint doi: https://doi.org/10.64898/2026.09.11.750342; this version posted September 13, 2026. The copyright holder for this preprint (which was not certified by peer review) is the author/funder, who has granted bioRxiv a license to display the preprint in perpetuity. It is made available under a CC-BY 4.0 International license.

Figure S3. Extended marker visualization across clusters.
Dot plot showing filtered marker sets for each cluster, including top-ranked differentially expressed genes and curated anchor markers used during cluster annotation. Genes expressed in less than 10% of cells in the target cluster were excluded from the visualization for display purposes only. Dot size represents the fraction of cells expressing each gene within a cluster, and color intensity reflects average expression.

bioRxiv preprint doi: https://doi.org/10.64898/2026.09.11.750342; this version posted September 13, 2026. The copyright holder for this preprint (which was not certified by peer review) is the author/funder, who has granted bioRxiv a license to display the preprint in perpetuity. It is made available under a CC-BY 4.0 International license.

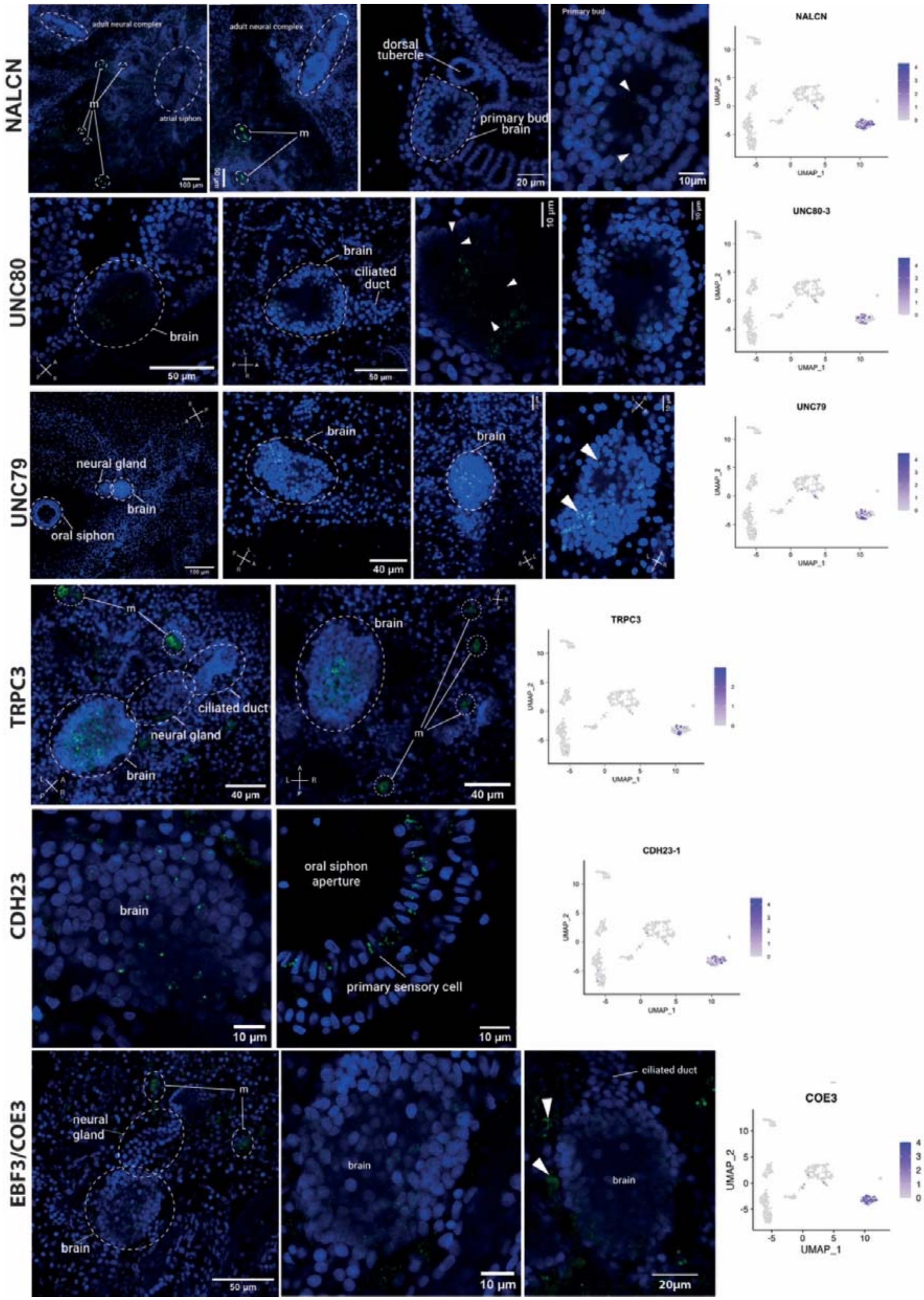

Figure S4A. Extended HCR-FISH validation of cluster 2-associated genes. Representative HCR-FISH images and corresponding UMAP feature plots for selected genes associated with the neuronal cluster of the Smart-seq3 adult neural-complex atlas, including NALCN, UNC80-3, UNC79, TRPC3, CDH23-1, and EBF3/COE3. For each gene,

bioRxiv preprint doi: https://doi.org/10.64898/2026.09.11.750342; this version posted September 13, 2026. The copyright holder for this preprint (which was not certified by peer review) is the author/funder, who has granted bioRxiv a license to display the preprint in perpetuity. It is made available under a CC-BY 4.0 International license.

the UMAP feature plot shows normalized expression across all cells in the single-cell dataset, with color intensity indicating log-normalized expression levels, whereas the HCR-FISH images show the spatial distribution of the corresponding transcripts in adult zooids and developing buds. Green indicates HCR-FISH signal and blue indicates Hoechst/DAPI nuclear counterstain. Dashed outlines indicate anatomical structures, including the brain, neural gland, ciliated duct, dorsal tubercle, primary bud brain, and oral siphon when visible. Arrowheads indicate HCR-positive cells or expression domains. Scale bars are indicated in each panel.

NALCN, UNC80-3, and UNC79 show enrichment in the neuronal cluster in the single-cell atlas and localize to sparse cells associated with the adult brain, neural-complex-adjacent territories, and developing bud neural structures. NALCNsignal is also detected in the developing primary bud brain. UNC80-3 and UNC79 are detected in cells within or adjacent to the adult brain and nearby neural-complex-associated domains, consistent with a neuronal/excitability-related identity.

TRPC3 is enriched in the neuronal cluster and is detected in cells within the adult brain as well as in adjacent neural-complex-associated territories, including the neural gland/ciliated duct region, with additional scattered mantle-associated cells (m) surrounding the neural complex.

CDH23-1 is enriched in the neuronal cluster and is detected both in the adult brain region and in peripheral sensory-associated cells around the oral siphon aperture, including primary sensory cells.

EBF3/COE3 is enriched in the neuronal cluster and is detected in the adult brain and in additional cells associated with the neural gland/ciliated duct region and surrounding territories, consistent with a broader neuronal differentiation-associated expression domain.

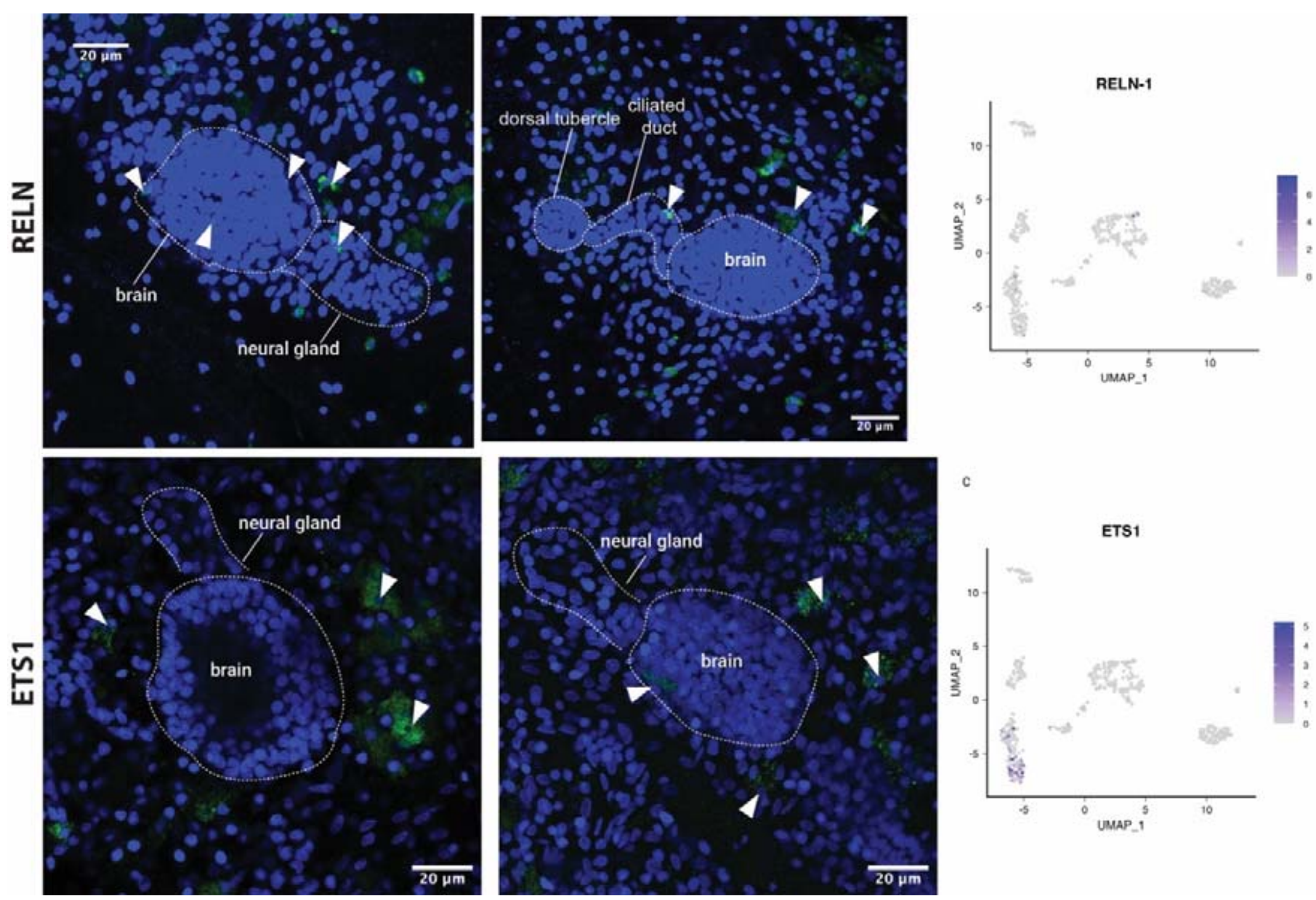

Figure S4B. Extended HCR-FISH validation of cluster 1-associated genes. Representative HCR-FISH images and corresponding UMAP feature plots for RELN-1 and ETS1, two genes associated with cluster 1 in the Smart-seq3 single-cell atlas. For each gene, the UMAP feature plot shows normalized expression across all cells of the adult *Botryllus schlosseri* neural-complex dataset, with color intensity indicating log-normalized expression levels. HCR-FISH images show the spatial distribution of the corresponding

bioRxiv preprint doi: https://doi.org/10.64898/2026.09.11.750342; this version posted September 13, 2026. The copyright holder for this preprint (which was not certified by peer review) is the author/funder, who has granted bioRxiv a license to display the preprint in perpetuity. It is made available under a CC-BY 4.0 International license.

transcripts in adult zooids and neural-complex-associated territories. RELN-1 signal is detected in sparse cells associated with the adult brain/neural gland region, the dorsal tubercle/ciliated duct area, and surrounding brain-adjacent tissues. ETS1 signal is detected in scattered cells surrounding the adult brain and neural gland region, as well as in additional cells distributed in adjacent adult zooid tissues. Green indicates HCR-FISH signal and blue indicates Hoechst/DAPI nuclear counterstain. Dashed outlines indicate anatomical structures, including the brain, neural gland, dorsal tubercle, and ciliated duct when visible. Arrowheads indicate HCR-positive cells or expression domains. Scale bars are indicated in each panel.

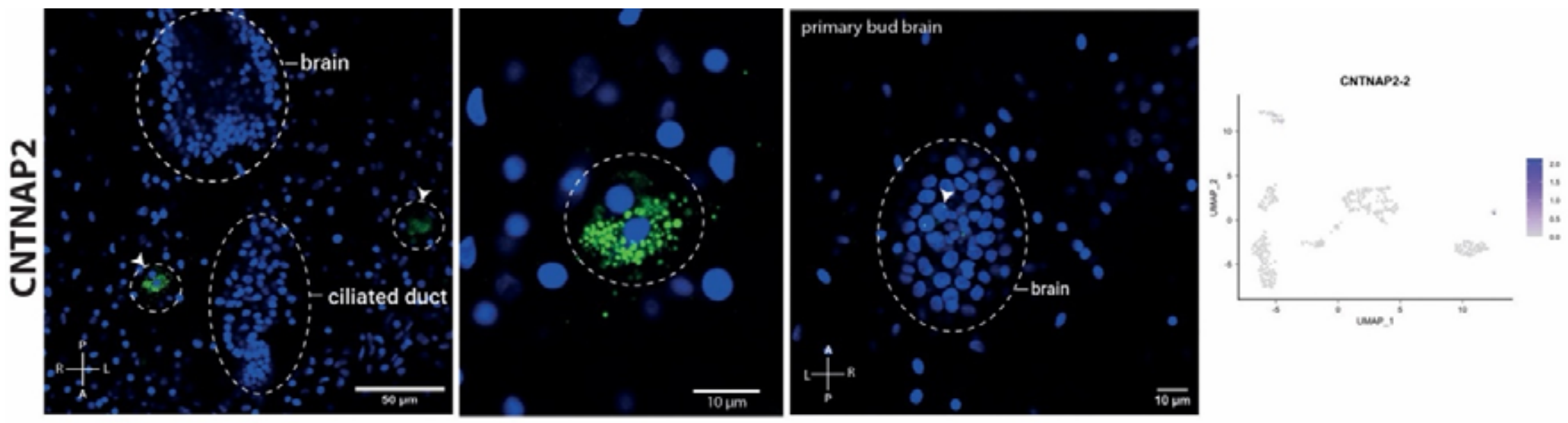

Figure S4C. HCR-FISH validation of CNTNAP2-2, cluster 4.
Representative HCR-FISH images and corresponding UMAP feature plot for CNTNAP2-2. In the adult zooid, CNTNAP2-2 signal is detected in discrete cell clusters located in mantle/perineural territories adjacent to the brain and ciliated duct. Higher magnification shows cytoplasmic CNTNAP2-2 signal in closely associated cells. In the primary bud, CNTNAP2-2-positive cells are detected in the developing bud brain. The paired UMAP feature plot shows restricted expression of CNTNAP2-2 in a small subset of cells in the Smart-seq3 atlas. Green, HCR-FISH signal; blue, DAPI/Hoechst nuclear counterstain. Dashed outlines indicate anatomical structures, and arrowheads indicate positive cells or expression domains. Scale bars are indicated in each panel.

bioRxiv preprint doi: https://doi.org/10.64898/2026.09.11.750342; this version posted September 13, 2026. The copyright holder for this preprint (which was not certified by peer review) is the author/funder, who has granted bioRxiv a license to display the preprint in perpetuity. It is made available under a CC-BY 4.0 International license.

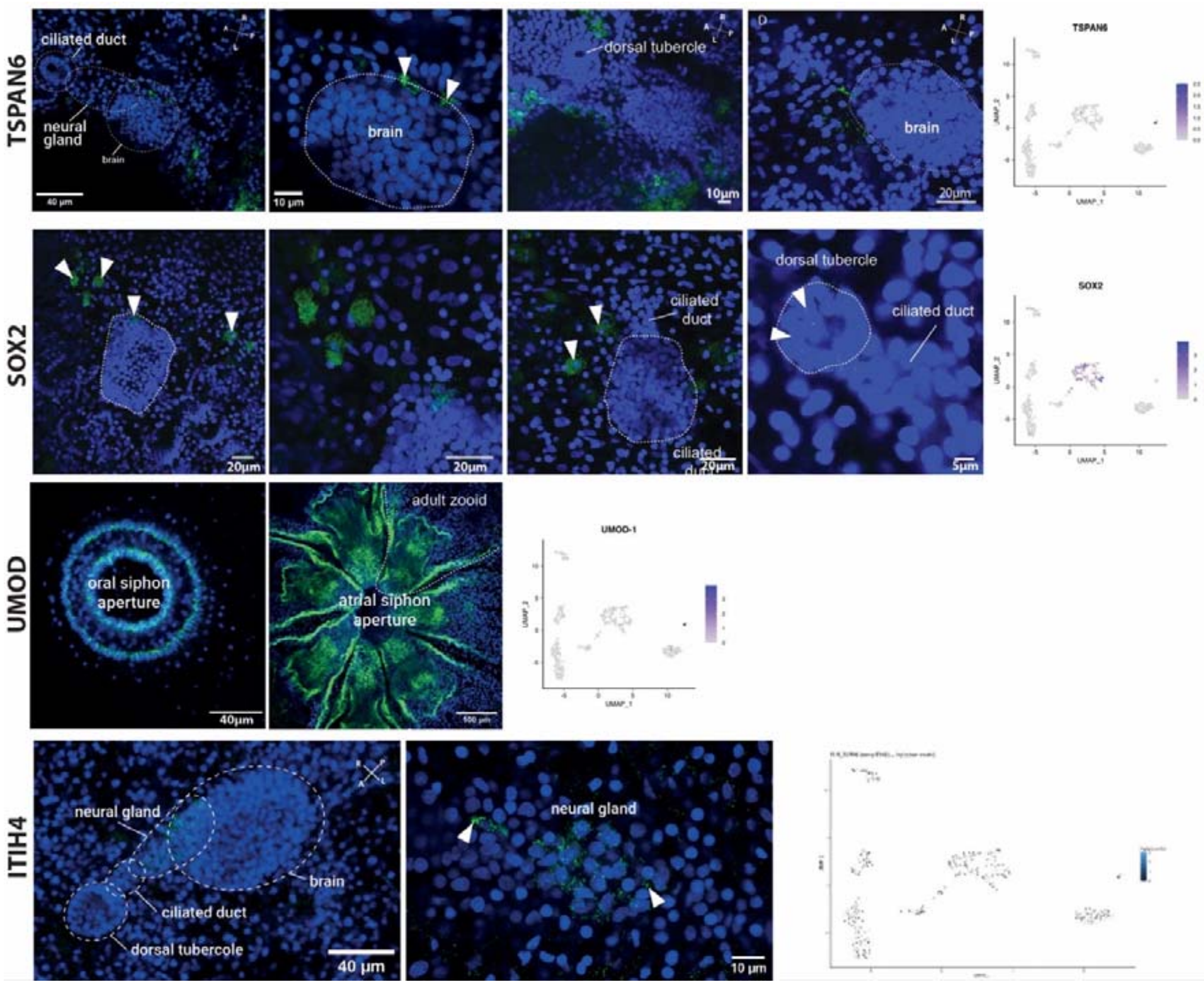

Figure S4D. Extended HCR-FISH validation of cluster 0-associated genes. Representative HCR-FISH images and corresponding UMAP feature plots for selected genes associated with the neuroepithelial/progenitor-like transcriptional programs, including TSPAN6, SOX2, UMOD-1, and ITIH4. For each gene, UMAP feature plots show normalized expression across the Smart-seq3 single-cell atlas, with color intensity indicating log-normalized expression levels. HCR-FISH images show the spatial distribution of the corresponding transcript in adult zooids and neural-complex-associated tissues. Green indicates HCR-FISH signal and blue indicates Hoechst or DAPI nuclear counterstain. Dashed outlines indicate anatomical structures, including the brain, neural gland, ciliated duct, and dorsal tubercle when visible. Arrowheads indicate HCR-positive cells or expression domains. Scale bars are indicated in each panel. TSPAN6 expression is detected in cells surrounding the adult brain and in adjacent perineural or mantle-associated territories, consistent with its expression in the single-cell atlas. SOX2, which is enriched in the neuroepithelial/progenitor-like cluster, is detected in cells surrounding the adult brain, in adjacent mantle/perineural territories, and weakly in the ciliated duct region. UMOD-1 shows limited expression in the single-cell atlas and spatially labels extra-brain epithelial territories, particularly siphonal domains including the oral and atrial siphon apertures. ITIH4 expression is detected in the neural gland region; higher magnification shows cytoplasmic ITIH4 signal in individual neural-gland-associated cells

bioRxiv preprint doi: https://doi.org/10.64898/2026.09.11.750342; this version posted September 13, 2026. The copyright holder for this preprint (which was not certified by peer review) is the author/funder, who has granted bioRxiv a license to display the preprint in perpetuity. It is made available under a CC-BY 4.0 International license.

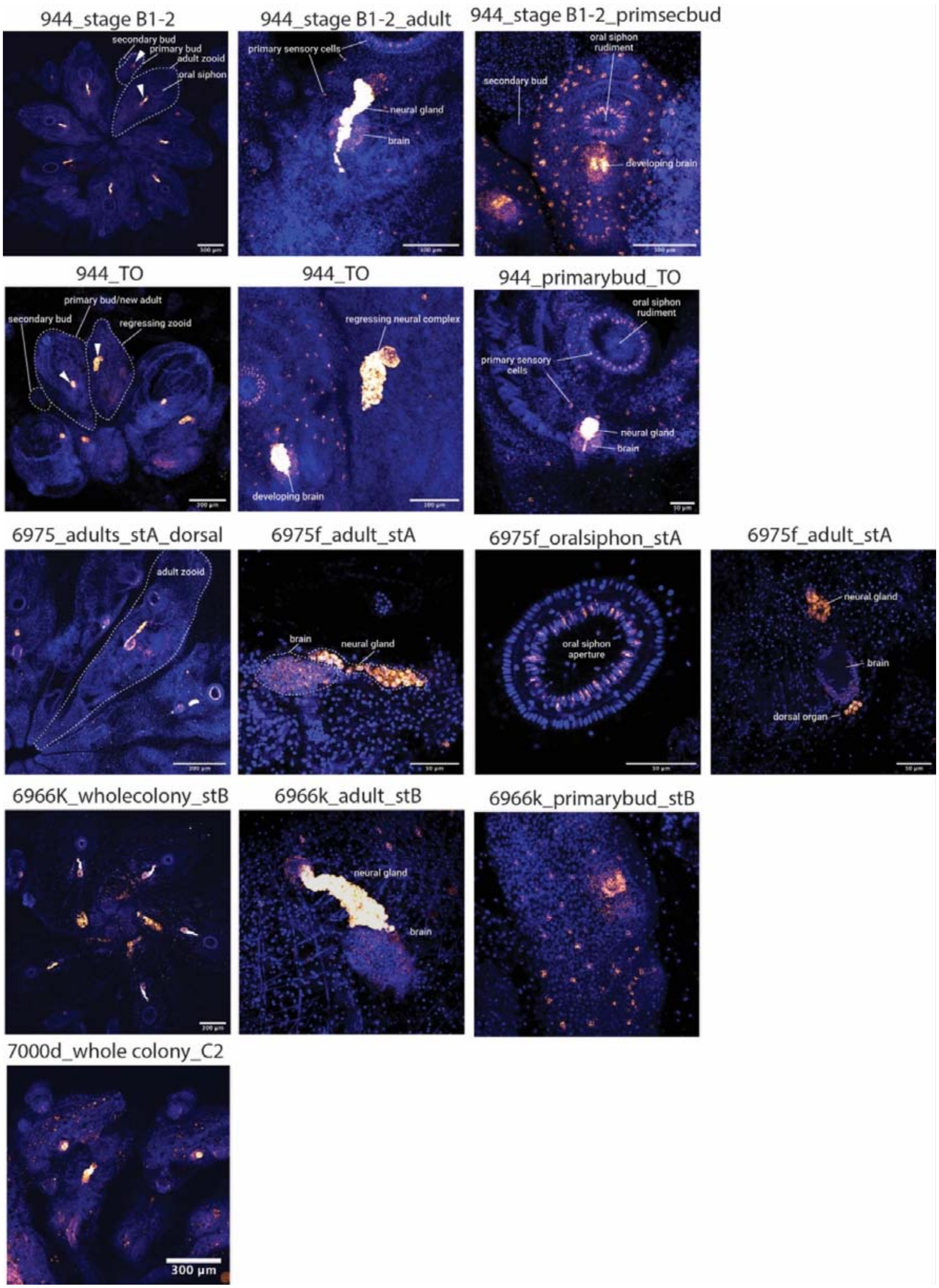

Figure S4E: Spatial expression of APP across blastogenetic stages and neural-complex-associated territories. HCR-FISH detection of APP transcripts reveals a broad and dynamic expression pattern across adult zooids and developing buds. In stage B colonies, APP signal is detected in the neural complex of the adult zooid as well as in the developing brain of the primary bud. During takeover (TO), APP expression is observed in

bioRxiv preprint doi: https://doi.org/10.64898/2026.09.11.750342; this version posted September 13, 2026. The copyright holder for this preprint (which was not certified by peer review) is the author/funder, who has granted bioRxiv a license to display the preprint in perpetuity. It is made available under a CC-BY 4.0 International license.

both the regressing adult neural complex and in the developing neural complex of the primary bud within the same colony, highlighting its presence across degenerative and developmental phases.In stage A adults, APP expression is particularly prominent in the neural gland and ciliated duct, where signal intensity exceeds that observed in the cerebral ganglion, which remains positively labeled but at lower levels. Across all stages, APP is consistently detected in primary sensory cells, corresponding to mechanoreceptive neurons distributed in the mantle epithelium and surrounding the oral siphon. At later stages (C2), APP expression is maintained in the neural complex of adult zooids as well as in primary and secondary buds.

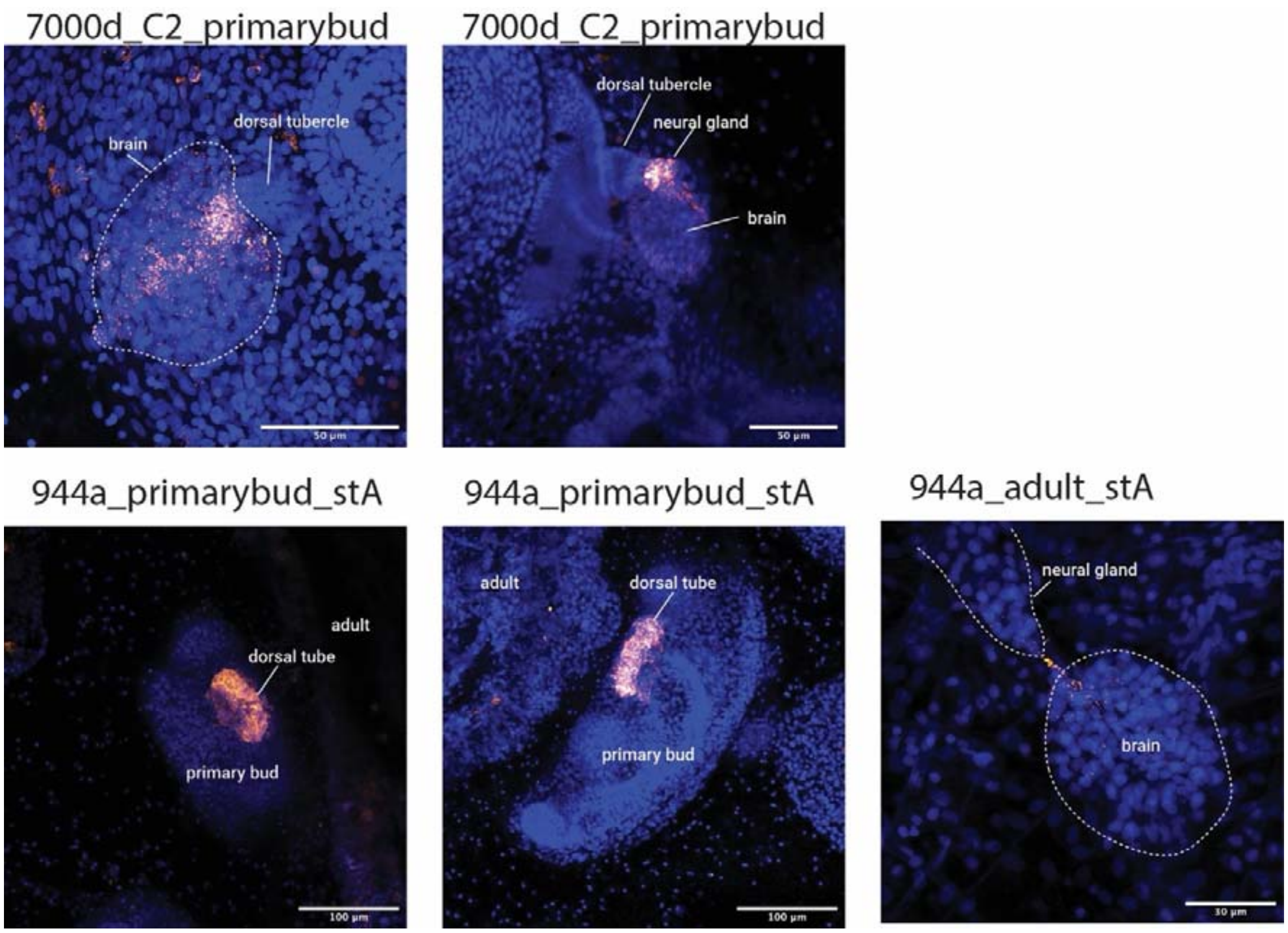

Figure S4F: Spatial expression of NEUROG1 in developing and adult neural-complex-associated territories in *Botryllus schlosseri*. HCR-FISH detection of NEUROG1 reveals a stage-dependent and spatially restricted expression pattern associated with neural-complex development. In primary buds at stage C2, NEUROG1 expression is detected within the neural complex, with stronger signal in the neural gland region compared to the developing cerebral ganglion. At earlier stages (stage A), NEUROG1 prominently marks the dorsal tube of the primary bud, a transient neurogenic structure that gives rise to both the neural gland and the cerebral ganglion. In adult zooids (stage A), NEUROG1 expression is markedly reduced, with only weak signal detected in the cerebral ganglion and a small number of positive cells located between the brain and the neural gland.

bioRxiv preprint doi: https://doi.org/10.64898/2026.09.11.750342; this version posted September 13, 2026. The copyright holder for this preprint (which was not certified by peer review) is the author/funder, who has granted bioRxiv a license to display the preprint in perpetuity. It is made available under a CC-BY 4.0 International license.

Figure S5E. Summary of HCR-FISH localization patterns across the adult neural complex

A **Anatomical summary**

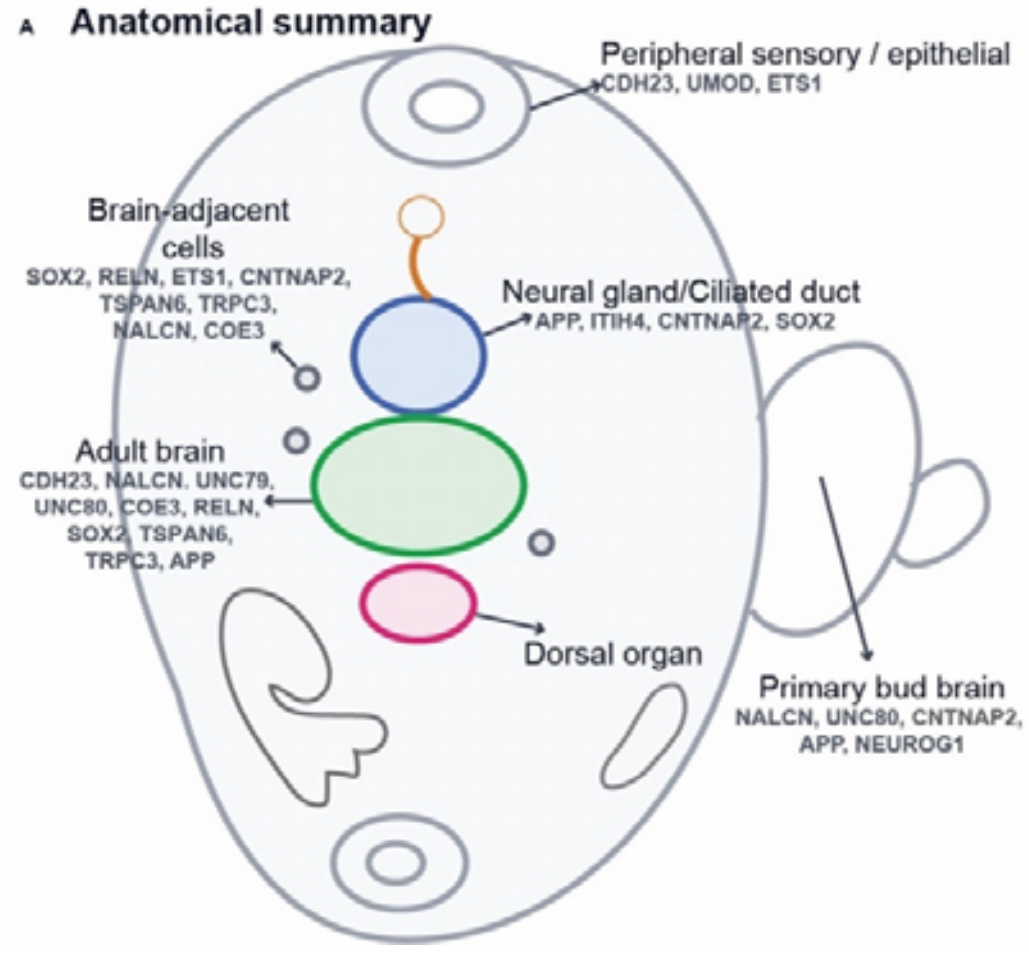

B **Gene × territory summary matrix**

Gene	Adult Brain	Brain-adjacent mantel	Primary bud brain	Neural gland / ciliated duct	Peripheral sensory / epithelial
**Brain-associated neuronal markers**					
CDH23	++	–	–	–	++
NALCN	+	+	++	–	–
UNC80	++	–	+	–	–
UNC79	++	–	–	–	–
COE3	+	+	–	–	–
TRPC3	++	+	–	–	–
**Brain-adjacent / progenitor-like / mantle-associated**					
RELN	rare	+	–	–	–
CNTNAP2	–	++	–	rare	–
SOX2	+	+	–	rare	–
ETS1	–	+	–	–	–
TSPAN6	+	++	–	–	–
**Neural gland / peripheral markers**					
APP	+	–	+	++	–
ITIH4	–	–	–	++	–
UMOD	–	–	–	–	++
NEUROG1	rare	–	++	+	–

Symbols: ++ strong; + detectable; rare sparse; – not detected.

Figure S4G. Summary of HCR-FISH localization patterns across the adult *B. schlosseri* neural complex.
(A) Schematic overview of the principal anatomical territories labeled by selected HCR-FISH markers in the adult zooid and neural-complex-associated region. Marker expression resolves multiple spatial categories, including adult brain-associated neuronal domains, brain-adjacent mantle-associated territories, neural gland/ciliated duct-associated domains, primary bud neural territories, and peripheral sensory/epithelial regions. (B) Matrix summarizing the predominant localization pattern of each HCR marker across major anatomical domains. Symbols indicate strong (++), detectable (+), sparse (rare), or undetected (**–**) signal in each territory.

bioRxiv preprint doi: https://doi.org/10.64898/2026.09.11.750342; this version posted September 13, 2026. The copyright holder for this preprint (which was not certified by peer review) is the author/funder, who has granted bioRxiv a license to display the preprint in perpetuity. It is made available under a CC-BY 4.0 International license.

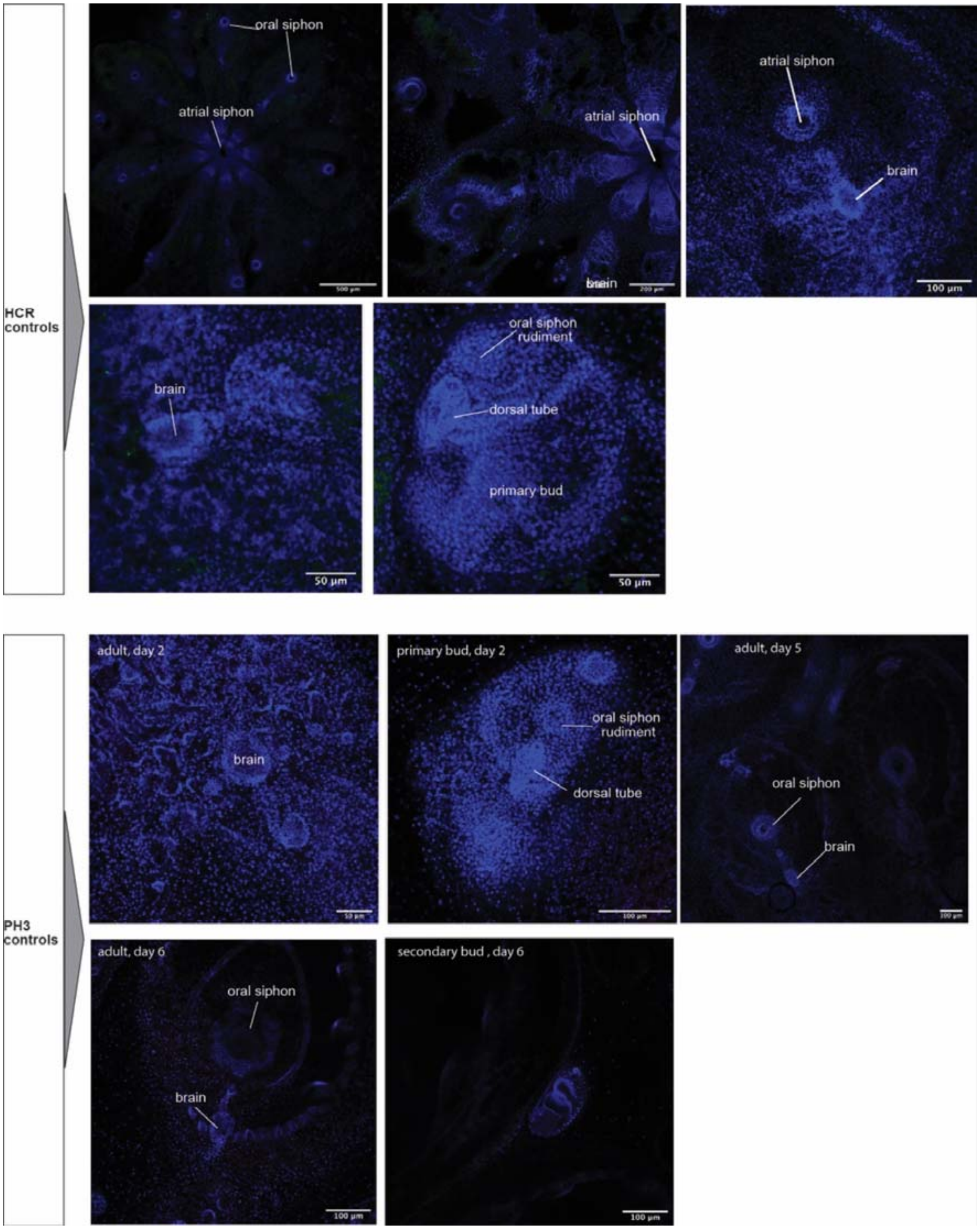

Figure S5. Negative controls for HCR-FISH and PH3 immunostaining.
Representative control images showing background signal in the absence of specific HCR probe signal or PH3 immunoreactivity. The upper panels show HCR-FISH control samples across adult zooids and developing buds, including views of the adult brain, atrial and oral siphons, and the primary bud dorsal tube. The lower panels show PH3 immunostaining controls in adult zooids and buds at different days of the blastogenetic cycle, including adult Day 2, primary bud Day 2, adult Day 5, adult Day 6, and secondary bud Day 6. Nuclei are counterstained with DAPI in blue. Anatomical landmarks are indicated to show the position

bioRxiv preprint doi: https://doi.org/10.64898/2026.09.11.750342; this version posted September 13, 2026. The copyright holder for this preprint (which was not certified by peer review) is the author/funder, who has granted bioRxiv a license to display the preprint in perpetuity. It is made available under a CC-BY 4.0 International license.

of the brain/neural complex, oral and atrial siphons, oral siphon rudiment, dorsal tube, and buds.. Scale bars are indicated in each image.

bioRxiv preprint doi: https://doi.org/10.64898/2026.09.11.750342; this version posted September 13, 2026. The copyright holder for this preprint (which was not certified by peer review) is the author/funder, who has granted bioRxiv a license to display the preprint in perpetuity. It is made available under a CC-BY 4.0 International license.

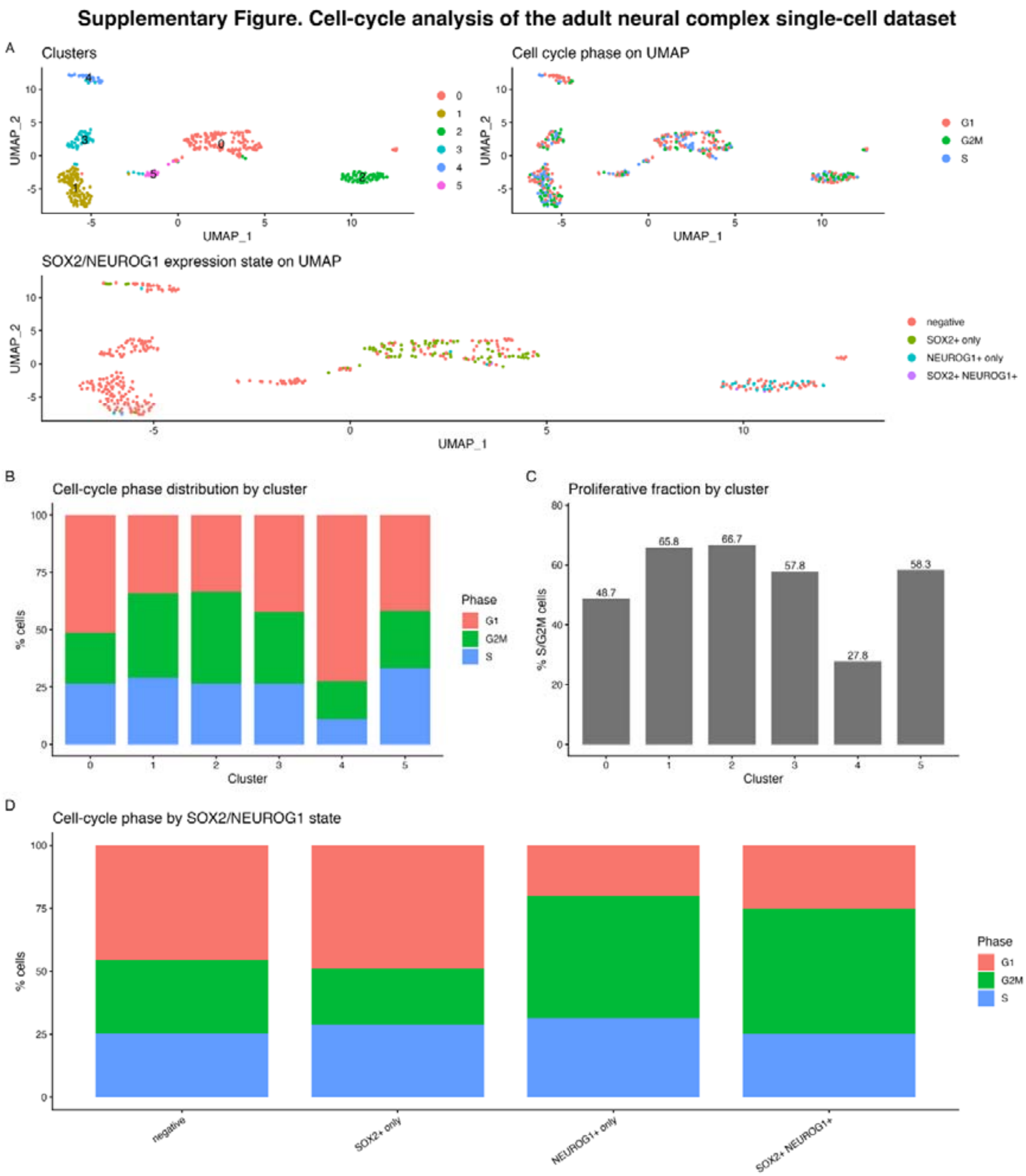

Figure S6. Cell-cycle phase distribution across clusters.
(A) UMAP representation of the single-cell dataset showing cluster identity, cell-cycle phase assignment, and SOX2/NEUROG1 expression state. SOX2-positive cells are mainly localized in cluster 0, whereas NEUROG1-positive cells are concentrated in cluster 2.
(B) Distribution of cells assigned to G1, S, or G2/M phases across clusters.
(C): Proliferative fraction by cluster, calculated as the percentage of cells assigned to S or G2/M phases. Clusters 1 and 2 showed the highest S/G2/M fractions, whereas cluster 4 showed the lowest proliferative fraction.
(D): Cell-cycle phase distribution stratified by SOX2/NEUROG1 expression state. SOX2-only cells showed a mixed G1 and S/G2/M profile, while NEUROG1-only cells were predominantly assigned to S/G2/M phases. SOX2/NEUROG1 double-positive cells were rare and should be interpreted cautiously. Overall, these analyses support the presence of actively cycling progenitor-like and neurogenic/committed precursor-like populations within the adult neural complex.

bioRxiv preprint doi: https://doi.org/10.64898/2026.09.11.750342; this version posted September 13, 2026. The copyright holder for this preprint (which was not certified by peer review) is the author/funder, who has granted bioRxiv a license to display the preprint in perpetuity. It is made available under a CC-BY 4.0 International license.

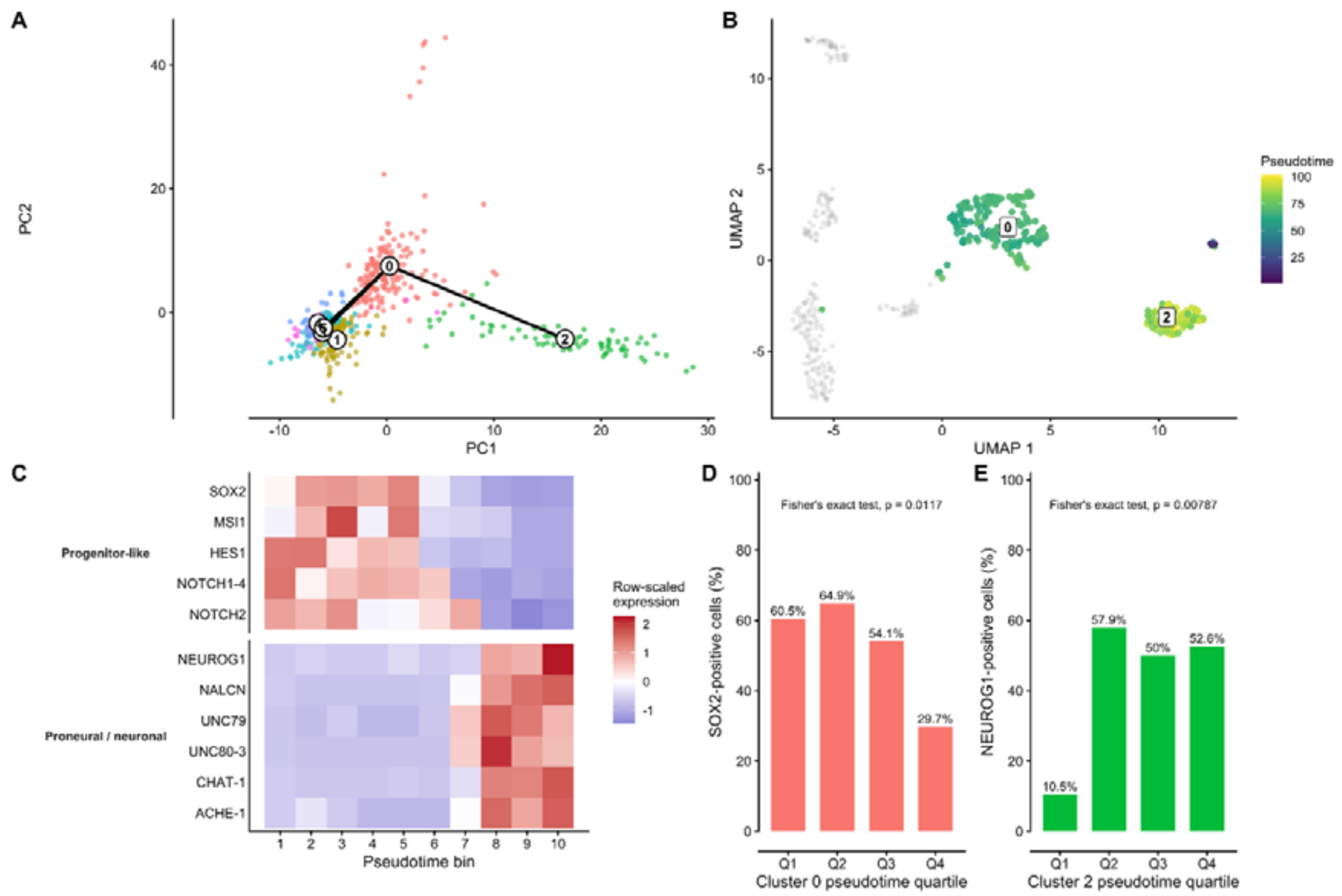

Figure S6B. Trajectory inference identifies a transcriptional axis linking progenitor-like and neuronal states.

(A) Unrooted Slingshot trajectory inference based on the first five principal components of the single-cell dataset. The cluster-level topology is displayed in the PC1–PC2 projection and directly connects progenitor-like cluster 0 with neuronal cluster 2 before specification of a starting state.

(B) Pseudotime along the inferred lineage connecting clusters 0 and 2 after cluster 0 was specified as a biologically informed starting state. Pseudotime was inferred in five-dimensional PCA space and projected onto the existing UMAP for visualization. The lineage contained 149 of 154 cluster 0 cells and all 75 cluster 2 cells; cells not assigned to this lineage are shown in gray.

(C) Expression dynamics of selected progenitor-associated and proneural/neuronal genes along the inferred 0–2 transcriptional axis. Cells were divided into ten equal-frequency pseudotime bins, mean normalized expression was calculated within each bin, and expression values were scaled independently for each gene across bins.

(D) Fraction of *SOX2*-positive cells within cluster 0 across pseudotime quartiles. SOX2-positive cells represented 60.5%, 64.9%, 54.1%, and 29.7% of cells in Q1–Q4, respectively (n = 38, 37, 37, and 37). SOX2 positivity differed significantly across quartiles (Fisher's exact test, p = 0.0117). Pairwise proportion tests with Benjamini–Hochberg correction showed significant reductions in Q4 relative to Q1 (adjusted p = 0.043) and Q2 (adjusted p = 0.031).

(E) Fraction of *NEUROG1*-positive cells within cluster 2 across pseudotime quartiles. *NEUROG1*-positive cells represented 10.5%, 57.9%, 50.0%, and 52.6% of cells in Q1–Q4, respectively (n = 19, 19, 18, and 19). *NEUROG1* positivity differed

bioRxiv preprint doi: https://doi.org/10.64898/2026.09.11.750342; this version posted September 13, 2026. The copyright holder for this preprint (which was not certified by peer review) is the author/funder, who has granted bioRxiv a license to display the preprint in perpetuity. It is made available under a CC-BY 4.0 International license.

significantly across quartiles (Fisher's exact test, $p = 0.00787$). Pairwise proportion tests with Benjamini–Hochberg correction showed significantly lower *NEUROG1* positivity in Q1 than in Q2 (adjusted $p = 0.037$), Q3 (adjusted $p = 0.047$), and Q4 (adjusted $p = 0.044$).

bioRxiv preprint doi: https://doi.org/10.64898/2026.09.11.750342; this version posted September 13, 2026. The copyright holder for this preprint (which was not certified by peer review) is the author/funder, who has granted bioRxiv a license to display the preprint in perpetuity. It is made available under a CC-BY 4.0 International license.

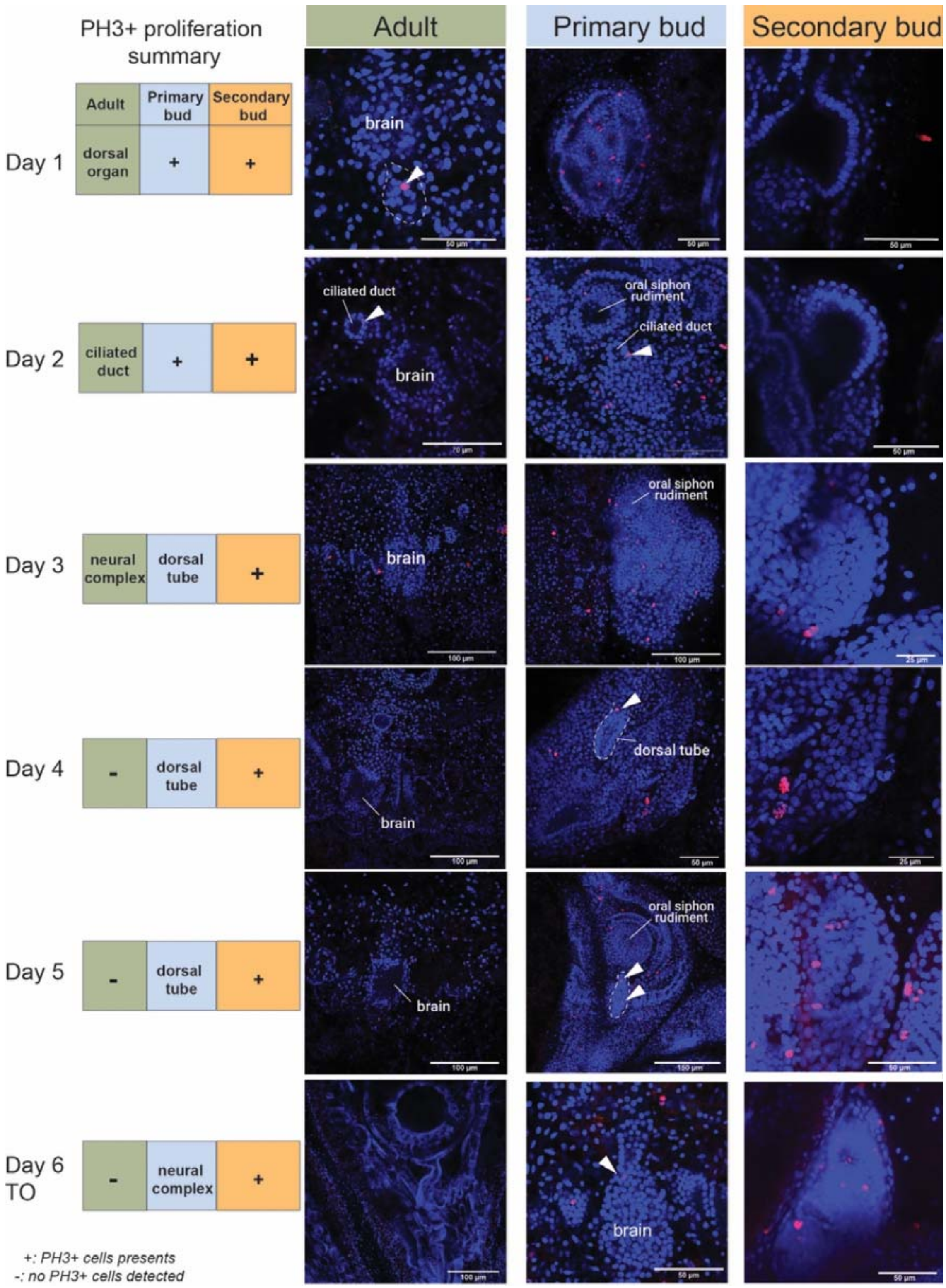

Figure S7: Stage-dependent PH3 labeling in the neural complex of adult and developing zooids across the blastogenetic cycle. Representative confocal images of

bioRxiv preprint doi: https://doi.org/10.64898/2026.09.11.750342; this version posted September 13, 2026. The copyright holder for this preprint (which was not certified by peer review) is the author/funder, who has granted bioRxiv a license to display the preprint in perpetuity. It is made available under a CC-BY 4.0 International license.

phospho-Histone H3 (PH3) immunostaining across successive blastogenetic stages, showing the adult, primary bud, and secondary bud neural regions from Day 1 to Day 6/takeover (TO). PH3-positive cells are detected in red and nuclei are counterstained in blue. The summary table on the left indicates, for each stage and generation, the anatomical region in which PH3+ cells were detected when this could be assigned with confidence. In the adult, PH3+ cells are observed mainly at early stages and are associated with the dorsal organ or ciliated duct region, whereas they are absent at intermediate late stages. In buds, proliferative cells are detected throughout development, although in early stages the signal cannot always be assigned unambiguously to a defined neural substructure. "+" indicates the presence of PH3+ cells without clear anatomical assignment; "−" indicates no PH3+ cells detected. Together, these data support persistent proliferative activity in developing buds and a more spatially restricted, stage-dependent pattern in the adult neural complex.

bioRxiv preprint doi: https://doi.org/10.64898/2026.09.11.750342; this version posted September 13, 2026. The copyright holder for this preprint (which was not certified by peer review) is the author/funder, who has granted bioRxiv a license to display the preprint in perpetuity. It is made available under a CC-BY 4.0 International license.

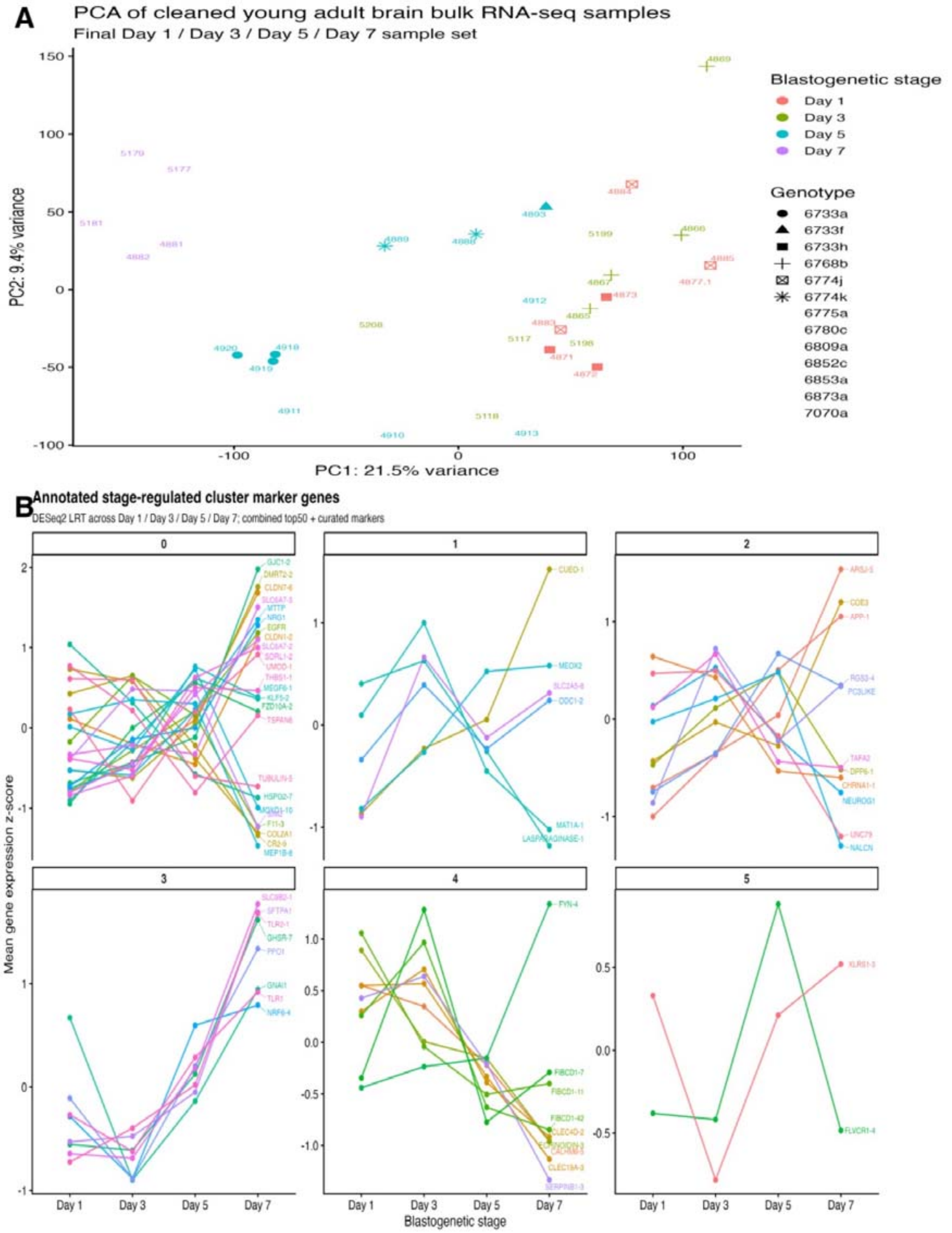

bioRxiv preprint doi: https://doi.org/10.64898/2026.09.11.750342; this version posted September 13, 2026. The copyright holder for this preprint (which was not certified by peer review) is the author/funder, who has granted bioRxiv a license to display the preprint in perpetuity. It is made available under a CC-BY 4.0 International license.

Figure S8. Additional bulk RNA-seq analyses across the blastogenetic cycle. (A) Principal component analysis of the cleaned young adult *Botryllus schlosseri* brain bulk RNA-seq dataset. Samples correspond to Day 1, Day 3, Day 5, and Day 7 of the blastogenetic cycle, corresponding to stages A, B, C, and D, respectively. Each point represents one bulk brain sample. Colors indicate blastogenetic stage and symbols indicate genotype. The PCA shows the global transcriptional structure of the cleaned dataset after removal of low-quality or misclassified samples. Later-stage samples, particularly Day 5 and Day 7, show partial separation from early Day 1/Day 3 samples along the first two principal components, consistent with stage-associated transcriptional variation.

(B) Annotated stage-regulated marker genes grouped by single-cell-derived cluster. Genes were selected by intersecting bulk stage-regulated genes identified by DESeq2 likelihood-ratio testing across Day 1, Day 3, Day 5, and Day 7 with the combined single-cell marker list. Only annotated genes were retained for visualization. Each line represents one gene, shown as mean z-scored expression across stages. Gene labels are shown at the final stage to facilitate identification of the major stage-associated trends. This panel provides an expanded view of the cluster-associated genes that vary significantly across the blastogenetic cycle.

bioRxiv preprint doi: https://doi.org/10.64898/2026.09.11.750342; this version posted September 13, 2026. The copyright holder for this preprint (which was not certified by peer review) is the author/funder, who has granted bioRxiv a license to display the preprint in perpetuity. It is made available under a CC-BY 4.0 International license.

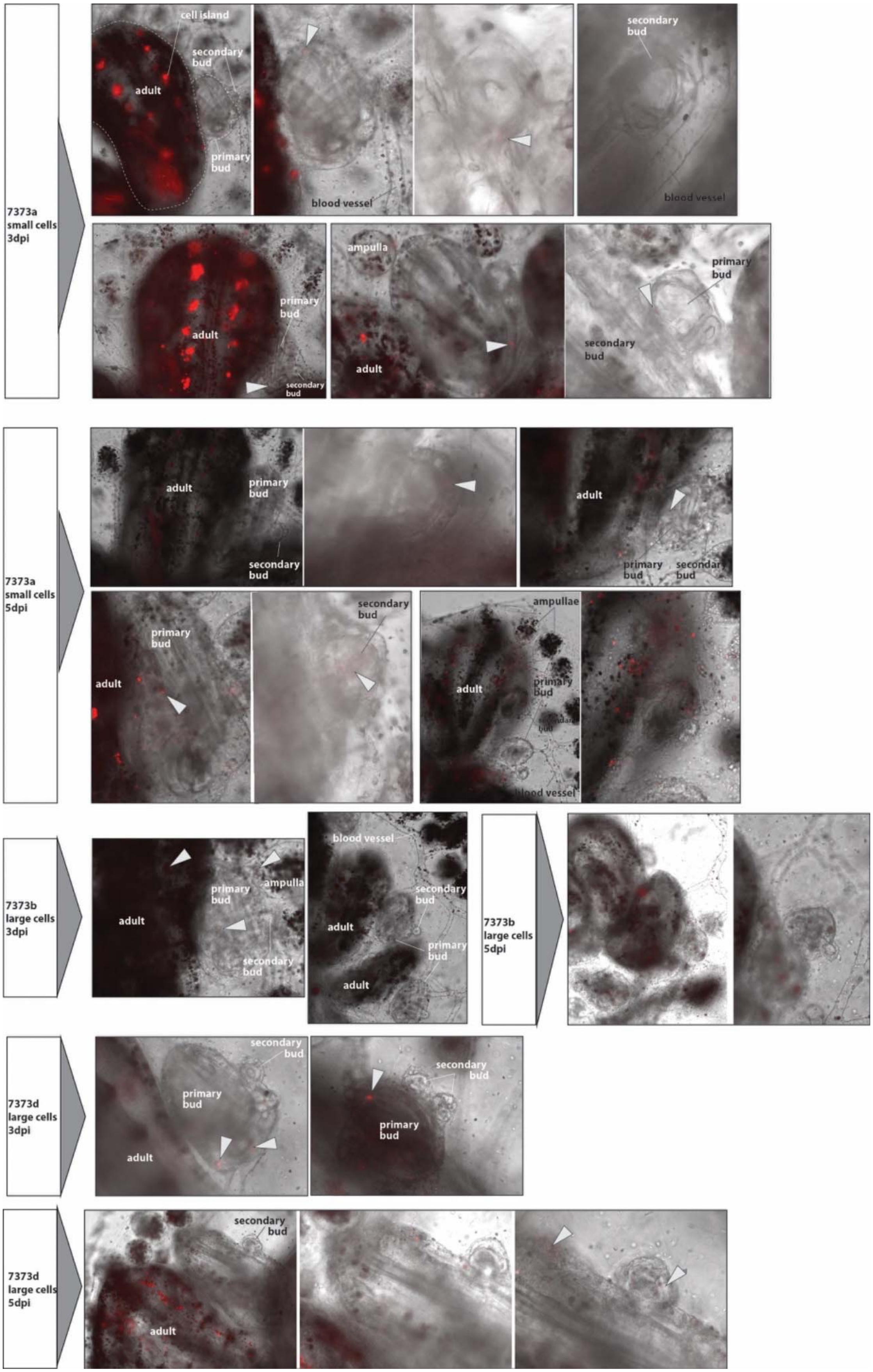

bioRxiv preprint doi: https://doi.org/10.64898/2026.09.11.750342; this version posted September 13, 2026. The copyright holder for this preprint (which was not certified by peer review) is the author/funder, who has granted bioRxiv a license to display the preprint in perpetuity. It is made available under a CC-BY 4.0 International license.

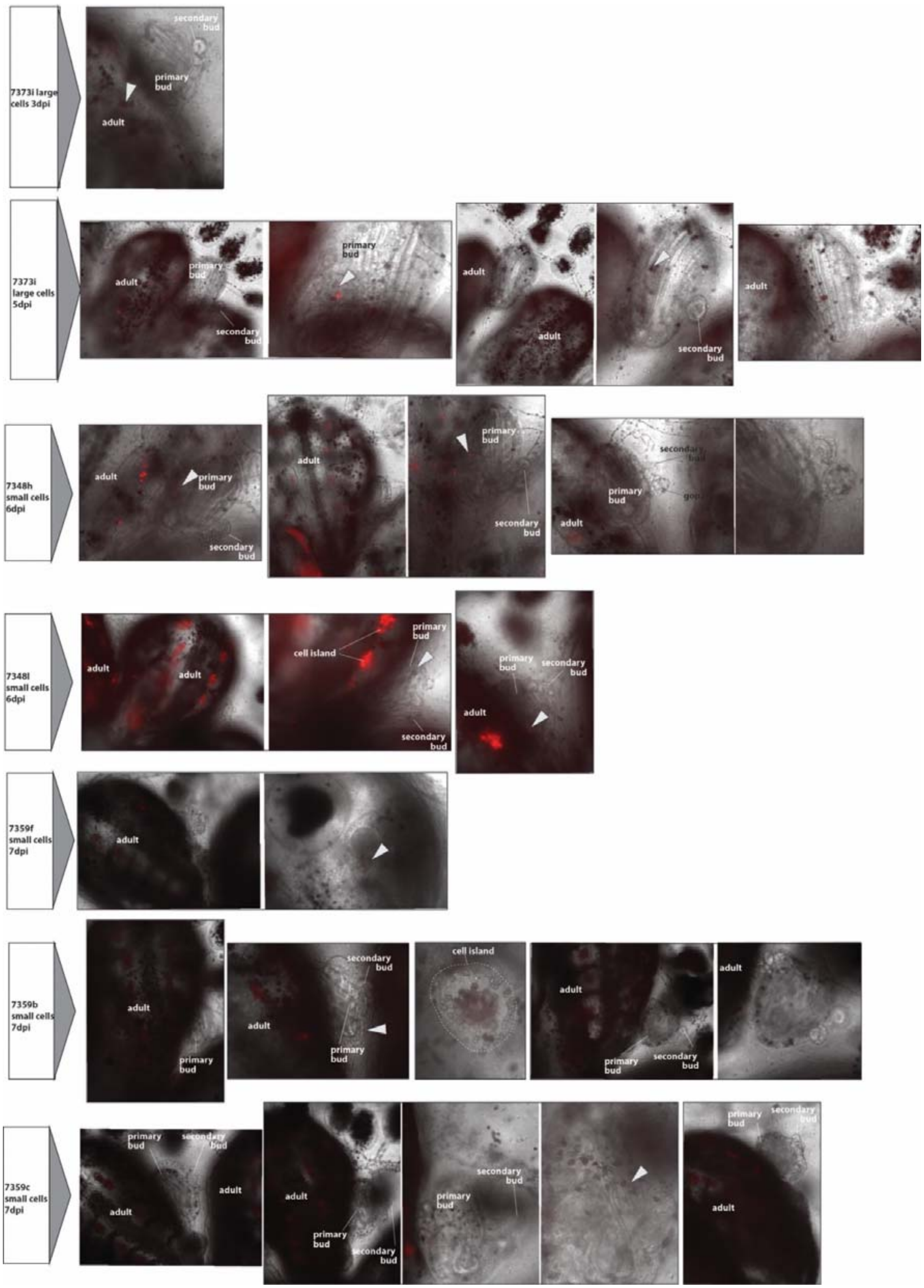

Figure S9. *In vivo* tracking of DiD-labelled cells following transplantation into recipient *Botryllus schlosseri*colonies. Representative live-imaging fields documenting the distribution of fluorescently labelled cells after injection of FACS-isolated small- or large-cell fractions into the extracorporeal vasculature of recipient colonies. DiD fluorescence is shown

bioRxiv preprint doi: https://doi.org/10.64898/2026.09.11.750342; this version posted September 13, 2026. The copyright holder for this preprint (which was not certified by peer review) is the author/funder, who has granted bioRxiv a license to display the preprint in perpetuity. It is made available under a CC-BY 4.0 International license.

in red and is overlaid on transmitted-light images to visualize the position of labelled cells relative to the adult zooid, primary and secondary buds, peripheral ampullae, blood vessels, endostyle-associated regions, gonadal territories, and circulating or tissue-associated cell islands. Images are grouped according to the injected cell fraction and post-injection observation time, as defined in the experimental dataset. White arrowheads indicate representative DiD-positive cells or cell aggregates, while dashed outlines identify selected anatomical structures or cell islands where shown. Labelled cells were detected in adult zooids and developing buds, as well as in the shared vascular system and peripheral ampullae, with substantial variability among colonies, anatomical compartments, and imaging fields. The full image series is provided to document the range of localization patterns observed across the analysed specimens, including both positive and signal-negative fields. Because DiD is a lipophilic membrane dye that can be progressively diluted during cell division and reduced through membrane turnover, later observations reflect persistence of detectable fluorescence rather than an absolute measurement of transplanted-cell survival, abundance, proliferation, or differentiation.

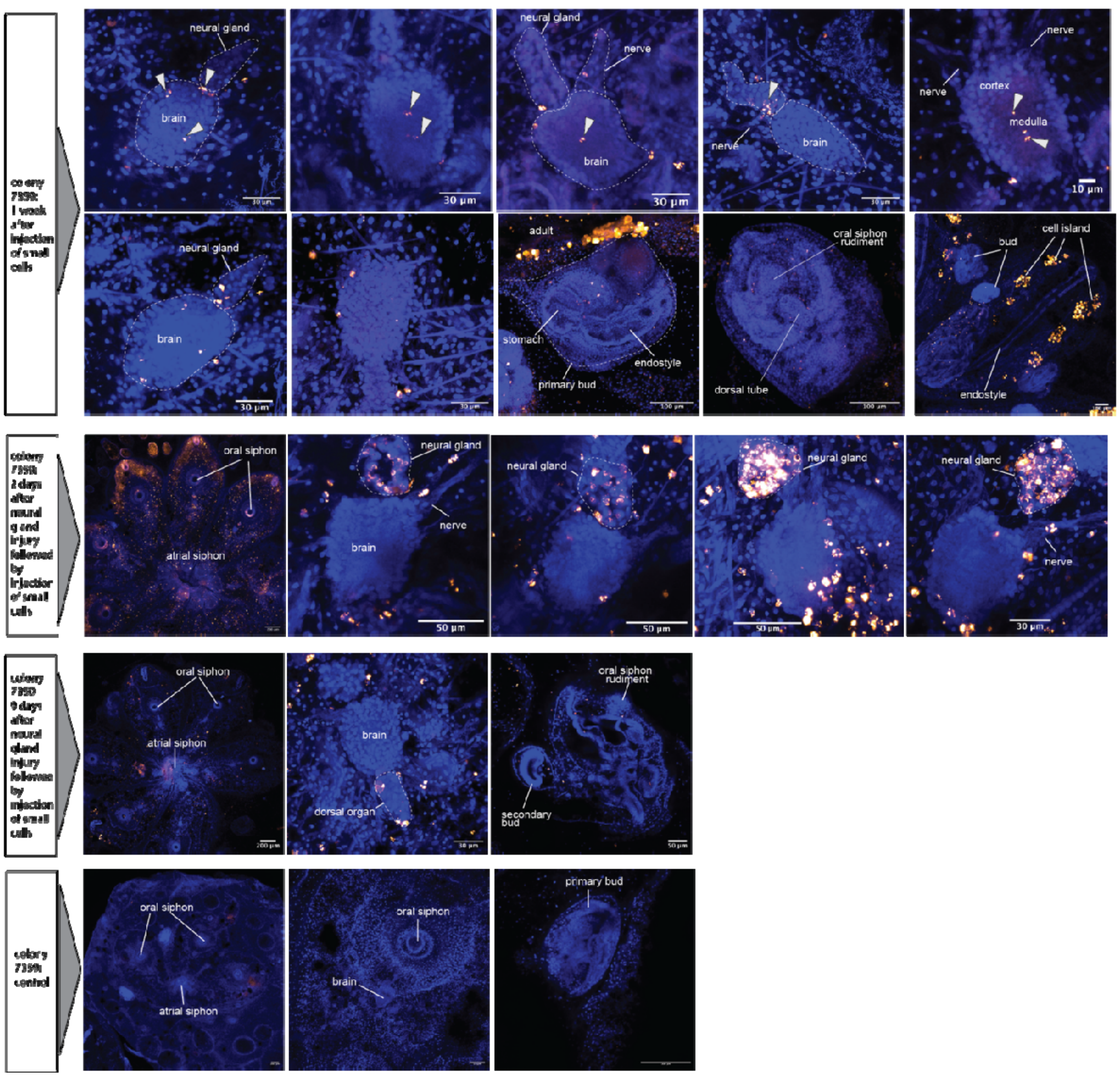

Figure S10:Confocal tracking of injected DiD-labelled small cells in adult and developing neural-complex-associated territories. Representative confocal images showing the distribution of DiD-labelled small cells after in vivo transplantation into recipient

bioRxiv preprint doi: https://doi.org/10.64898/2026.09.11.750342; this version posted September 13, 2026. The copyright holder for this preprint (which was not certified by peer review) is the author/funder, who has granted bioRxiv a license to display the preprint in perpetuity. It is made available under a CC-BY 4.0 International license.

Botryllus schlosseri colonies. Nuclei are shown in blue and DiD-labelled cells or cell aggregates are shown in red/orange. In uninjured colonies examined approximately one week after injection, labelled cells were detected within and around the adult brain, at the brain–neural gland interface, along peripheral nerve-associated regions, and in developing bud-associated territories. Dashed outlines indicate the approximate boundaries of the brain and neural gland where shown, and arrowheads indicate representative DiD-positive cells or aggregates. Additional whole-zooid and bud-level views document labelled cells in adult tissues, the primary bud, the secondary bud, dorsal tube-associated regions, and cell-island/endostyle-associated territories. Following targeted injury to the neural gland region, numerous DiD-labelled cells were observed around the neural gland and adjacent brain one day after injury. Labelled cells remained detectable in neural-complex-associated regions nine days after injury, including around the neural gland, brain, and nearby nerves. Images are representative of the available confocal dataset and were included to document the range of anatomical localizations observed across specimens. Scale bars are indicated in each panel. Because DiD fluorescence can be progressively diluted during cell division and can decrease with membrane turnover over time, the images indicate persistence of detectable label rather than an absolute measure of transplanted-cell survival, proliferation, or integration.
